# Goiter and hearing impairment: A case of a male patient with Pendred syndrome

**DOI:** 10.3892/ol.2014.2461

**Published:** 2014-08-19

**Authors:** ER-WEI HU, LI-BIN LIU, RUO-YU JIANG, XIANG-HUI HE

**Affiliations:** Department of General Surgery, Tianjin Medical University General Hospital, Heping, Tianjin 300052, P.R. China

**Keywords:** sensorineural deafness, goiter, Pendred syndrome, thyroid dysfunction

## Abstract

Pendred syndrome is a rare genetic disease that causes a disturbance in thyroid hormone synthesis, which results in thyroid dysfunction and the development of goiter and sensorineural deafness. The present report describes the case of a young euthyroid male, who developed a large goiter and hearing impairment, despite no family history of these conditions. A left lobectomy and a subtotal right lobectomy were performed, and the patient was administered permanent hormone replacement therapy with thyroxine. Patients with Pendred syndrome exhibit distinct clinical features and the mechanisms associated with the molecular genetics of this disease have been clarified. Thus, gene detection is considered to be a reliable diagnostic method. Certain patients require surgical intervention in order to relieve the symptoms. Misdiagnosis can be significantly reduced by increasing the understanding of Pendred syndrome.

## Introduction

Pendred syndrome, also termed goiter-deafness syndrome, is a relatively rare recessive genetic disease, which is characterized by congenital deafness and progressive clinical enlargement of a goiter. Pendred syndrome was first recognized by Vaughan Pendred, a British physician, in 1896 (1). A single mutant recessive gene, SLC26A4(PDS), which encodes the protein pendrin, is considered to be responsible for the goiter and deafness. Previous studies have indicated that this protein functions as a chloride/iodine pump (2). A lack of awareness of this disease in clinical practice often results in cases of misdiagnosis or missed diagnoses. Thus, the details of a typical patient with Pendred syndrome are presented. Clinicians must raise awareness regarding the disease and ensure correct diagnosis and treatment.

The present report discusses a patient with a goiter and a hearing impairment, expounding the characteristics of Pendred syndrome so as to raise the awareness of the disease and ensure the correct diagnosis and treatment. The patient provided written informed consent.

## Case report

A 26-year-old male presented to the Tianjin Medical University General Hospital (Tianjin, China) due to a painless lump in the neck, which had been inadvertently identified 6 years previously. The lump had recently begun to exhibit rapid growth and the patient reported symptoms of agitation, irritability, hyperhidrosis and discomfort whilst swallowing. Prior to presentation at Tianjin Medical University General Hospital (Heping, China), the patient had been diagnosed with nodular goiter by physicians at other hospitals. The patient had intermittently taken Euthyrox^®^ for numerous years. The patient reported a hearing impairment and learning difficulties with language in childhood, however, a clinical examination showed that his mental and physical development was normal.

**Figure 1:**
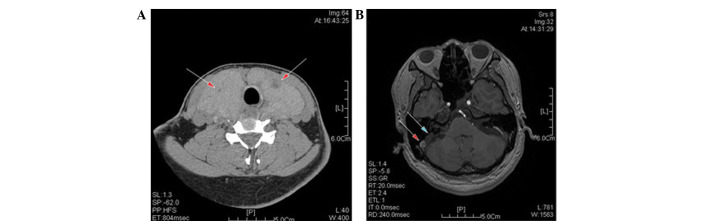
Thyroid ultrasound images. (A) Multiple low echo nodules in each lobe and isthmus of the thyroid (indicated by the arrows). (B) Enlargement of the vestibular aqueducts (indicated by the green arrow) and cavernous sinus (indicated by the red arrow).

**Figure 2:**
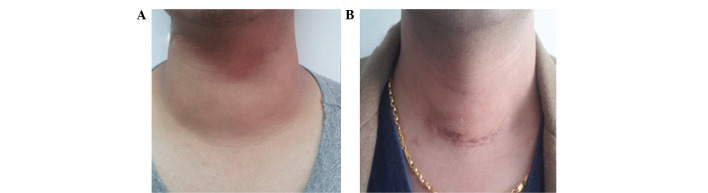
Images of the patient (A) prior to and (B) following the thyroidectomy.

**Figure 3:**
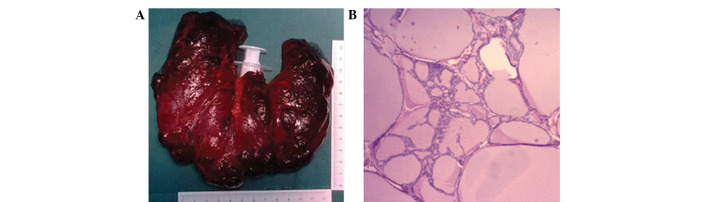
Isolated thyroid specimen following the thyroidectomy. (A) The two thyroid tissues measured ~15×13×7 cm, weighed 500 g, were dark red in color and contained colloid. (B) Microscopic pathological analysis showed that the thyroid follicular epithelial cells became short and the hyperplasia was reduced. The absorption cavitations decreased, and the quantity of colloid increased and became thicker. The capillary blood congestion was relieved between the interstitial spaces and the proportion of lymphocytes decreased. Magnification, ×40.

Physical examination showed a visible goiter, grade III according to the World Health Organisation and Pan American Health Organization criteria (3). The thyroid was enlarged, and felt tough with no palpable nodules and the thyroid boundary was clear. Laboratory tests revealed a free thyroxine level of 9.99 pmol/l (normal rannge, 11.5–23.5 pmol/l), a free triiodothyronine level of 6.07 pmol/l (normal range, 3.5–6.5 pmol/l) and a thyroid stimulating hormone level of 2.401 μlU/ml (0.3–5.0 μlU/ml). The thyrotrophin receptor, thyroglobulin and thyroperoxidase antibodies, carcinoembryonic antigen, thyroglobulin and calcitonin levels were within the normal ranges. A chest X-ray revealed a narrowing of the trachea at the level of the superior aperture of the thorax, and a thyroid ultrasound showed multiple hypoechoic signals in each lobe and isthmus of the thyroid. Furthermore, a cervical lymph node enlargement was revealed (Fig. 1). The manifestations of the cervical computed tomogrophy scan included a diffuse enlargement of each lobe, and a thickening of the isthmus of the thyroid gland. Numerous, oval, low- and slightly high-density nodules were identified in the thyroid. Electrical capacitance tomography results were consistent with the signs of a nodular goiter. Magnetic resonance imaging of the inner ear showed that the two vestibular aqueducts were enlarged, more markedly in the right aqueduct. The surgical procedure was performed under general anesthesia with an endotracheal intubation. A left lobectomy and a subtotal right lobectomy were performed and the patient experienced an uneventful postoperative period. Post surgery, the patient was administered permanent hormone replacement therapy with thyroxine (Fig. 2). The thyroid pathology following the thyroidectomy is shown in Fig. 3.

## Discussion

Pendred syndrome is an autosomal recessive disorder, which is characterized by familial goiter, congenital deafness and organic iodine deficiency disorders (4). The most prominent clinical symptom in patients with Pendred syndrome is bilateral congenital sensorineural deafness (5). The degree of deafness may differ among individual patients, and may manifest at birth or appear gradually (6). The majority of patients have a speech disorder, and a small number may experience tinnitus and vertigo (7). The relevant imaging examinations in patients with Pendred syndrome show that the majority of patients exhibit a vestibular aqueduct enlargement, with or without inner ear malformation. Furthermore, patients commonly present with a dilated lymph sac and lymphatic vessels (8). A typical inner ear deformation, termed Mondini malformation, is an abnormality of the cochlear that is characterized by an abnormally short and flat cochlear structure (9). Goiter is another feature of Pendred syndrome that typically develops in patients following the onset of deafness. The early stages of goiter is diffuse, which subsequently and gradually develops into multiple nodular goiter. The common clinical sign of goiter in young patients is diffuse enlargement. In adults, the thyroids are typically enlarged with multiple palpable nodules, but with no tremors or vascular murmurs. It has previously been reported that Pendred syndome has the potential to become cancerous (10). Furthermore, goiter is exhibited to varying degrees in patients with Pendred syndrome, however, it is most significant between the ages of 20 and 30 years (8). The molecular mechanisms of Pendred syndrome have been determined by previous studies. The pathogenic gene, SLC26A4 (also termed *PDS*) is located on chromosome 7q31 (11). The *PDS* gene encodes the pendrin protein, a highly hydrophobic transmembrane protein that consists of 780 amino acids (12). A previous study demonstrated that this protein is highly expressed in the thyroid, inner ear and kidney (13). Pendrin functions as a chloride/iodide pump in the thyroid, and is responsible for transporting iodine out of the cell and into the follicular colloid (2). A *PDS* gene mutation may cause abnormal protein expression and affect iodine transport. As a result, patients may develop a clinically enlarged thyroid. Pendrin is expressed in the inner ear for the transport of chloride-formate, which has a critical role in the maintenance of a stable endolymph environment. *PDS* mutations may, therefore, result in an abnormal pendrin structure and influence its chloride ion transport function in the inner ear. Furthermore, abnormal pendrin structure and function leads to an increase in the internal pressure of the vestibular aqueduct and lymphatic vessels, which increases the pressure of the internal ear. As a result, patients are clinically characterized with a hearing impairment and even deafness (14).

Pendred syndrome was first described by Pendred (1) in 1896. Brian *et al* reported the case of an inbred family from London, comprising of 12 siblings who were identified to suffer from the goiter-deafness syndrome (15). It was concluded from the observations that the disease may be associated with a recessive gene mutation (16). Despite previous studies, the majority of clinicians are unfamiliar with the disease, resulting in frequent misdiagnoses. Furthermore, patients with Pendred syndrome present with different clinical manifestations, including varying degrees of hearing impairment and thyroid dysfunction, which complicates diagnosis (Dhariry). The perchlorate discharge test has traditionally been used to diagnose Pendred syndrome, however, it is not a specific diagnostic test (17). Patients are administered 10 mg/kg perchlorate, which is taken orally, and Iodine-131 (^131^I) uptake rates are measured 1 h prior to and following administration. A positive test is indicated by an ^131^I uptake rate of >10%. The perchlorate discharge test facilitates the diagnosis of Pendred syndrome, however, it is not considered to be the gold standard. The majority of patients exhibit euthyroid goiter, however, certain patients exhibit signs of hypothyroidism (6). In the present case report, a patient with Pendred syndrome was presented, who had no family history of deafness or goiter. The patient had experienced progressive hearing damage since early childhood and, from adolescence, had gradually developed goiter and shown signs of hypothyroidism. Genetic testing is a specific diagnostic method for detecting *PDS* mutations, however, it is currently difficult to perform in clinical practice. As a result, clinical manifestations and imaging methods are more commonly adopted to determine the diagnosis. There is currently no effective treatment for Pendred syndrome. Adequate thyroid hormone replacement therapy should be prescribed early to prevent further development of goiter. In patients without oppressive symptoms and probable canceration, surgery is not recommended. However, in the present case the patient underwent a left lobectomy and a subtotal right lobectomy due to the large size of the goiter and tracheal compression, which was caused by rapid growth.

In order to manage the goiter of Pendred syndrome, patients require adequate assessment, including observation of clinical symptoms, as well as imaging examinations of the thyroid. Due to the persistence of pathogenic factors, surgical removal of sections of the gland may lead to goiter recurrence postoperatively. As aforemtioned, surgery must be avoided in patients without oppressive symptoms and suspicious canceration. In patients with the aforementioned symptoms surgical intervention is of great significance as due to the persistence of pathogenic factors the surgical removal of the gland may lead to postoperative goiter recurrence. Early intervention measures can be taken to prevent disease progression and reduce thyroid growth, thereby reducing the likelihood of obstruction and canceration.

In conclusion, it is important to determine an early diagnosis of Pendred syndrome; however, reducing the rate of missed diagnoses and misdiagnoses requires further investigation, thus, it is essential for clinicians to improve their understanding of Pendred syndrome. Misdiagnosis may be reduced, in part, by screening for suspected cases, obtaining a full report of the family history and performing relevant imaging examinations. The incidence of the disease may be reduced via genetic analysis of the disease-causing *PDS* gene, genetic counseling and eugenic prenatal detection. The clinical understanding of Pendred syndrome, as well as the molecular and genetic research based on clinical diagnosis, will have a vital role in the prevention, early detection, diagnosis and treatment of this disease.
